# Regulation of Cardiomyocyte T-Tubular Structure: Opportunities for Therapy

**DOI:** 10.1007/s11897-017-0329-9

**Published:** 2017-04-26

**Authors:** Ornella Manfra, Michael Frisk, William E. Louch

**Affiliations:** Institute for Experimental Medical Research, Oslo University Hospital and University of Oslo, Kirkeveien 166, NO-0407 Oslo, Norway

**Keywords:** T-tubules, Heart failure, Cardiomyocytes, Calcium homeostasis, Junctophilin-2, Bridging integrator-1

## Abstract

**Purpose of Review:**

Membrane invaginations called t-tubules play an integral role in triggering cardiomyocyte contraction, and their disruption during diseases such as heart failure critically impairs cardiac performance. In this review, we outline the growing understanding of the malleability of t-tubule structure and function, and highlight emerging t-tubule regulators which may be exploited for novel therapies.

**Recent Findings:**

New technologies are revealing the nanometer scale organization of t-tubules, and their functional junctions with the sarcoplasmic reticulum called *dyads*, which generate Ca^2+^ sparks. Recent data have indicated that the dyadic anchoring protein junctophilin-2, and the membrane-bending protein BIN1 are key regulators of dyadic formation and maintenance. While the underlying signals which control expression and localization of these proteins remain unclear, accumulating data support an important role of myocardial workload.

**Summary:**

Although t-tubule alterations are believed to be a key cause of heart failure, the plasticity of these structures also creates an opportunity for therapy. Promising recent data suggest that such therapies may specifically target junctophilin-2, BIN1, and/or mechanotransduction.

## Introduction

In mammalian cardiac myocytes, invaginations of the sarcolemmal membrane create an extensive network called the t-tubule system. These structures allow the cardiac action potential to propagate into the interior of the myocyte, initiating the process of excitation-contraction (EC) coupling. This role is afforded by precise control of the structure and function of t-tubules and their constituent ion transporters. Recent data have revealed an impressive malleability of t-tubules, giving new insight into how they are assembled and maintained, but also how they can be pathologically altered. In the present review, we outline these exciting new findings, and particularly highlight the emerging understanding of how t-tubule structure and function may be therapeutically targeted in diseases such as heart failure.

## T-Tubule Structure

**Fig. 1:**
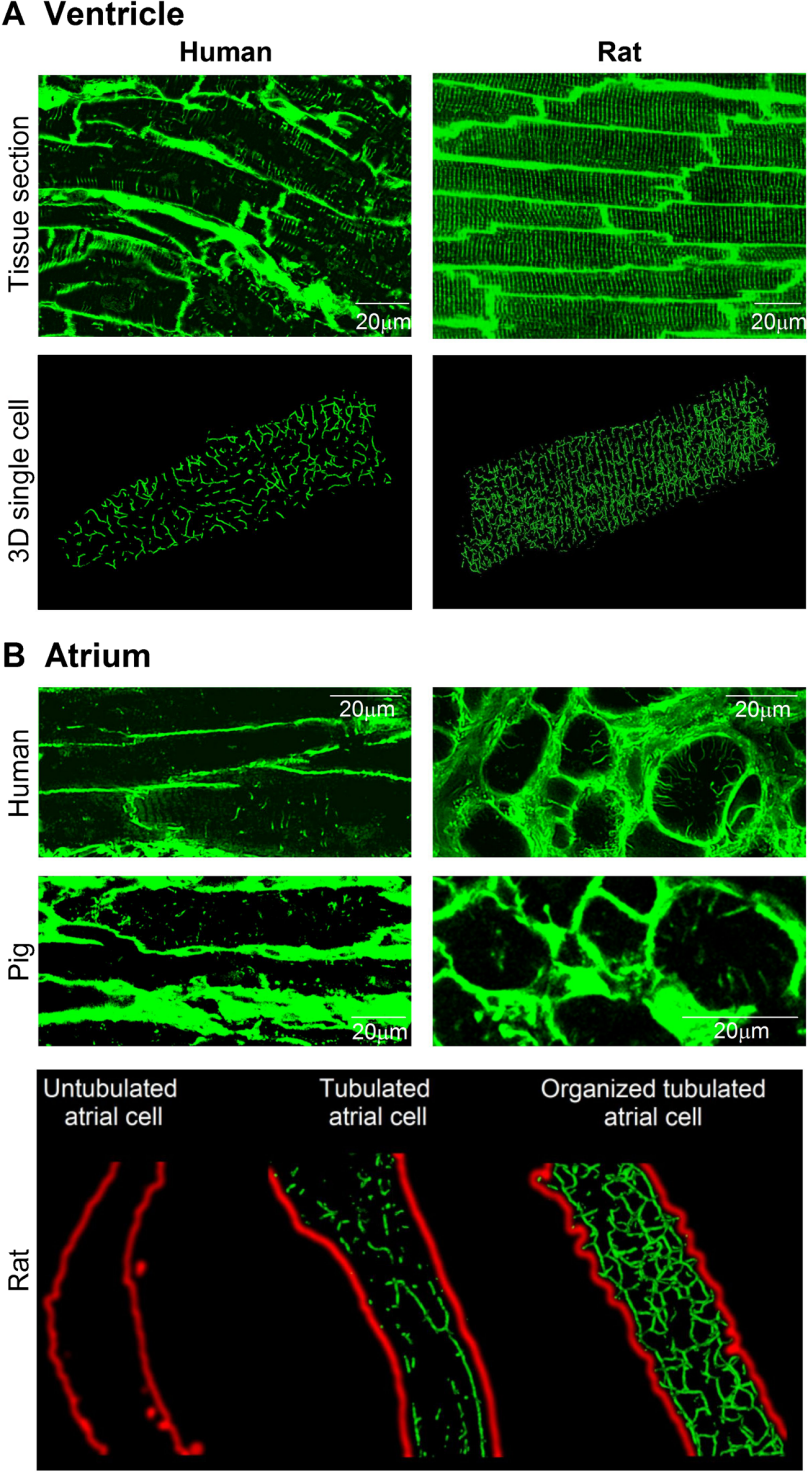
T-tubule organization in ventricular and atrial cardiac myocytes. **a** Confocal images of the t-tubule network in tissue sections from human ventricle (top left, unpublished) and rat ventricle (top right, modified from [3]), labeled with wheat germ agglutinin (WGA) and lipophilic membrane indicator FM4-64, respectively. Three-dimensional reconstructions of single cardiomyocytes from human and rat ventricle are shown in the lower panels (WGA labeling, human cell unpublished, rat cell reproduced from [4]) **b** Confocal images of the t-tubule network in tissue sections from human and pig atria (upper panels, WGA labeling; [4, 5]), and isolated rat atrial myocytes (lower panel, di-8-ANEPPS staining; [4]). Variable t-tubule organization was observed across the atria in all three species. Images from [3–5], reproduced with permissions

In ventricular cardiomyocytes, t-tubules are present in a well-organized network. The majority of tubules in these cells are oriented transversely along z-lines, leading to the original designation of this system as the “transverse” or “t”-tubule network [1, 2]. However, closer examination revealed a surprisingly high proportion of tubules which run along the longitudinal axis of the cell, forming roughly perpendicular junctions with their transversely-oriented counterparts (Fig. 1a) [6]. In an effort to more accurately describe this bi-directional structural arrangement, the network is sometimes referred to as the transverse-axial tubule system (TATS) [7]. In ventricular cardiomyocytes, t-tubules vary in diameter (20–450 nm [6]), constituting 0.8–3.6% of the total cell volume and 21–64% of the total sarcolemma [8–11]. Species-dependent differences also exist, with a tendency toward denser and thinner structures in smaller species with higher heart rates (Fig. 1a) [6, 12, 13].

T-tubules were long reported to be absent in atrial myocytes, and it was often hypothesized that the smaller, thinner size of these cells precluded a necessity for initiation of excitation-contraction coupling at internal sites [14]. Recent data have dispelled this belief, with numerous reports indicating the presence of t-tubules in both large and small mammalian species (Fig. 1b) [4, 5, 15–19]. However, in comparison with ventricular myocytes, the t-tubule network of atrial cells is generally less well developed, and more variable between cells. In rat, for example, we and others have observed that only approximately 1/3 of atrial cells are tubulated, and when present, the arrangement of tubules is often predominantly longitudinal (Fig. 1b) [4, 20].

## T-Tubule Function

**Fig. 2:**
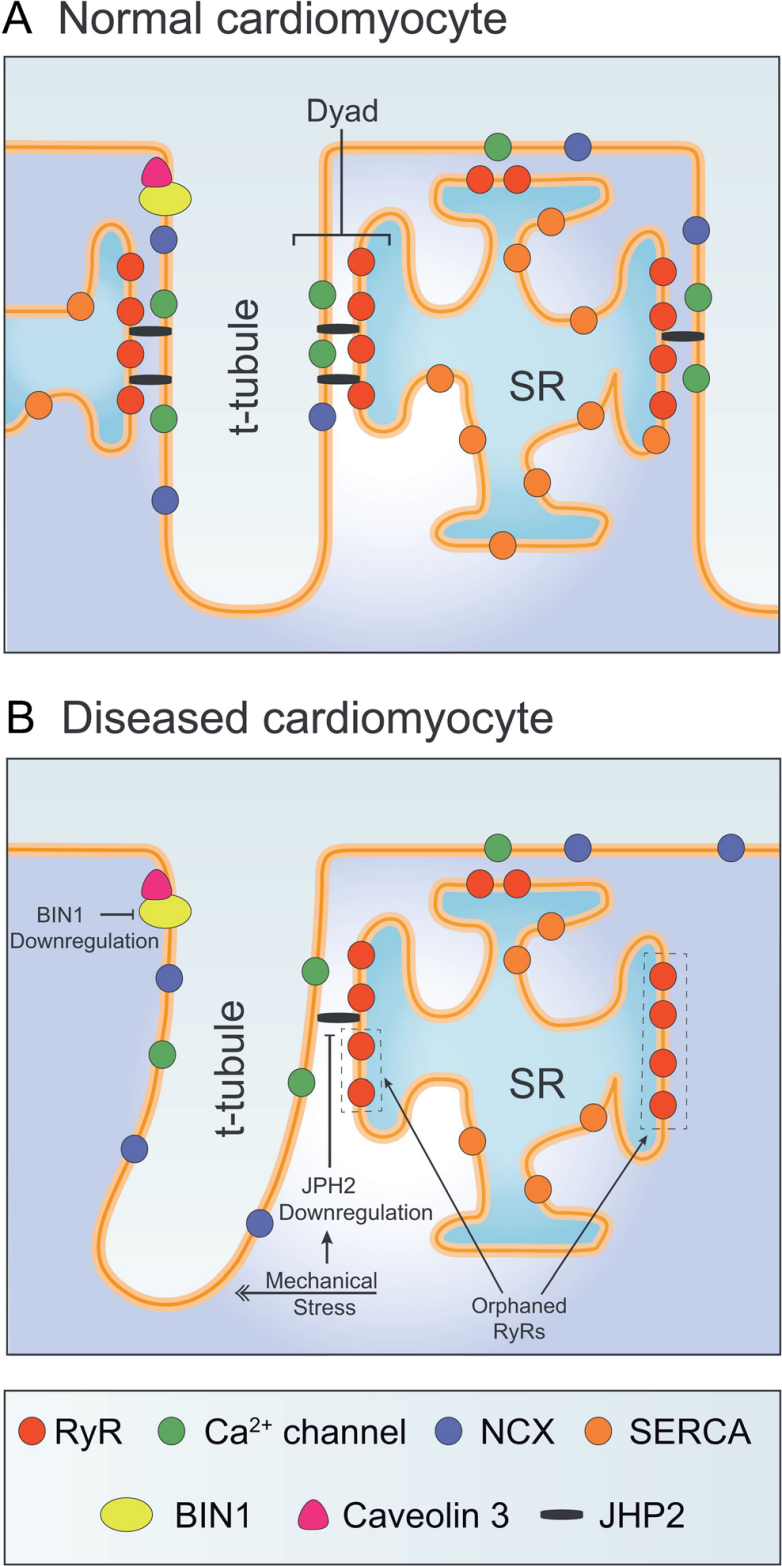
T-tubule structure in normal and failing cardiac myocytes. **a** In healthy cardiomyocytes, L-type Ca^2+^ channels in the t-tubules are apposed from ryanodine receptors (RyRs) in the sarcoplasmic reticulum (SR). Excitation-contraction coupling occurs at these dyadic junctions, which are maintained by Junctophilin-2 (JPH2) and BIN1, and dynamically regulated by workload. **b** Elevated workload during heart failure has been linked to downregulation of JPH2 and BIN1, and disorganization/loss of t-tubules. Such remodeling results in the formation of orphaned RyRs, which are uncoupled from Ca^2+^channels, reduced efficiency of Ca^2+^-induced Ca^2+^release, and impaired contractility in this condition. Novel opportunities for cellular-level heart failure therapies include mitigation of workload/mechanotransduction and strategies for elevating JPH2 and BIN1 expression

While the gross morphology of t-tubules in ventricular and atrial myocytes has been described in ever increasing detail, more attention has also been given to their nanometer scale organization and function. Of key importance for EC coupling are specialized junctions between the membranes of the t-tubules and sarcoplasmic reticulum (SR) known as *dyads* (Fig. 2). Within these couplings, which are located along both transverse and longitudinally-oriented t-tubules [21, 22], L-type Ca^2+^ channels (LTCCs) face ryanodine receptors (RyRs) in the SR membrane, across a narrow 12–15 nm dyadic cleft [23]. As the action potential is propagated into the t-tubules by the opening of Na^+^ channels, this depolarization triggers LTCC opening and the resulting Ca^2+^ entry elicits SR Ca^2+^ release via the RyRs. This process, known as Ca^2+^-induced Ca^2+^ release (CICR), results in high cytosolic Ca^2+^ concentration and initiation of contraction as Ca^2+^ binds to the myofilaments. Precise positioning of LTCCs and RyRs is essential for efficient CICR. Both proteins are present in clusters [24] with an estimated stoichiometry of 4–10 RyRs per LTCC [25]. In recent years, several studies have employed super-resolution microscopy to gain further insight into the arrangement of RyRs. In rat ventricular cardiomyocytes, Baddeley *et al.* reported that each dyad contains an average of 14 RyRs, grouped into a number of “super-clusters” [26]. Close localization of these super-clusters allows their cooperation in the production of a Ca^2+^ spark, the fundamental unit of Ca^2+^ release in cardiomyocytes [27]. Openings of individual super-clusters, on the other hand, have been suggested to underlie smaller release events known as Ca^2+^ quarks [28].

During relaxation of the cell, released Ca^2+^ is recycled into the SR by the action of SERCA and extruded from the cell by the Na^+^-Ca^2+^ exchanger in the cell membrane. Like LTCCs, NCX is expressed at higher density in the t-tubules than in the surface sarcolemma, and along both transverse and longitudinal tubules [22, 29]. Although its precise location has long been debated, there is a growing consensus that a significant fraction of NCX molecules are colocalized with RyRs, suggesting that they are located in or very near dyads (Fig. 2) [24, 30–32]. This localization is believed to ensure rapid Ca^2+^ removal following a Ca^2+^ spark [33]. However, when functioning in so-called “reverse mode”, NCX can yield Ca^2+^ influx which may trigger SR Ca^2+^ release from nearby RyRs [34–37]. Such activity is promoted by local elevation of dyadic Na^+^ levels and, since the NCX is electrogenic, by depolarized membrane potentials.

Even in the absence of trigger Ca^2+^ from nearby LTCCs or NCX, RyR Ca^2+^ release can be elicited by Ca^2+^ diffusion from neighboring dyads. In its most dramatic form, spontaneous Ca^2+^ waves of CICR can be observed to propagate across entire cardiomyocytes. However, in cells with low t-tubule density, smaller wave-like patterns of Ca^2+^ release also occur during the action potential, as Ca^2+^ diffuses from intact dyads into the gaps between them where non-dyadic or “orphaned” RyRs are present [38–43] (Fig. 2). Thus, t-tubule density is an important determinant of the synchrony of Ca^2+^ release across the cardiomyocyte, and thus the kinetics of the rising phase of the Ca^2+^ transient.

## Malleability of T-Tubule Structure/Function during Health and Disease

T-tubules exhibit remarkable plasticity of both their structure and function, and this has important consequences for Ca^2+^ homeostasis and overall cardiac function. In small rodents, t-tubules are absent in neonatal cardiomyocytes and start to appear as the heart matures. This growth starts with a rudimentary, largely longitudinally-oriented network, which then evolves into a denser, primarily transversely-oriented system [44]. Interestingly, a recent study reported that sheep exhibit earlier t-tubule development than small rodents, with appearance of t-tubules in utero [45], suggesting that there are significant species-dependent differences in the time course of maturation. Importantly, RyRs are localized at internal, rudimentary SR cisternae prior to the growth of t-tubules in developing cardiomyocytes [44, 46]. Thus, the action potential of immature cardiomyocytes induces a wave-like propagation of Ca^2+^ from the surface sarcolemma toward orphaned RyRs at the cell interior [47]. The appearance of t-tubules and assembly of LTCCs into dyads coincides with an augmenting role in NCX-mediated Ca^2+^ extrusion, and maturation of this adult-like mode of Ca^2+^ cycling corresponds temporally with strengthening of systole and diastole (reviewed in [48]).

**Fig. 3:**
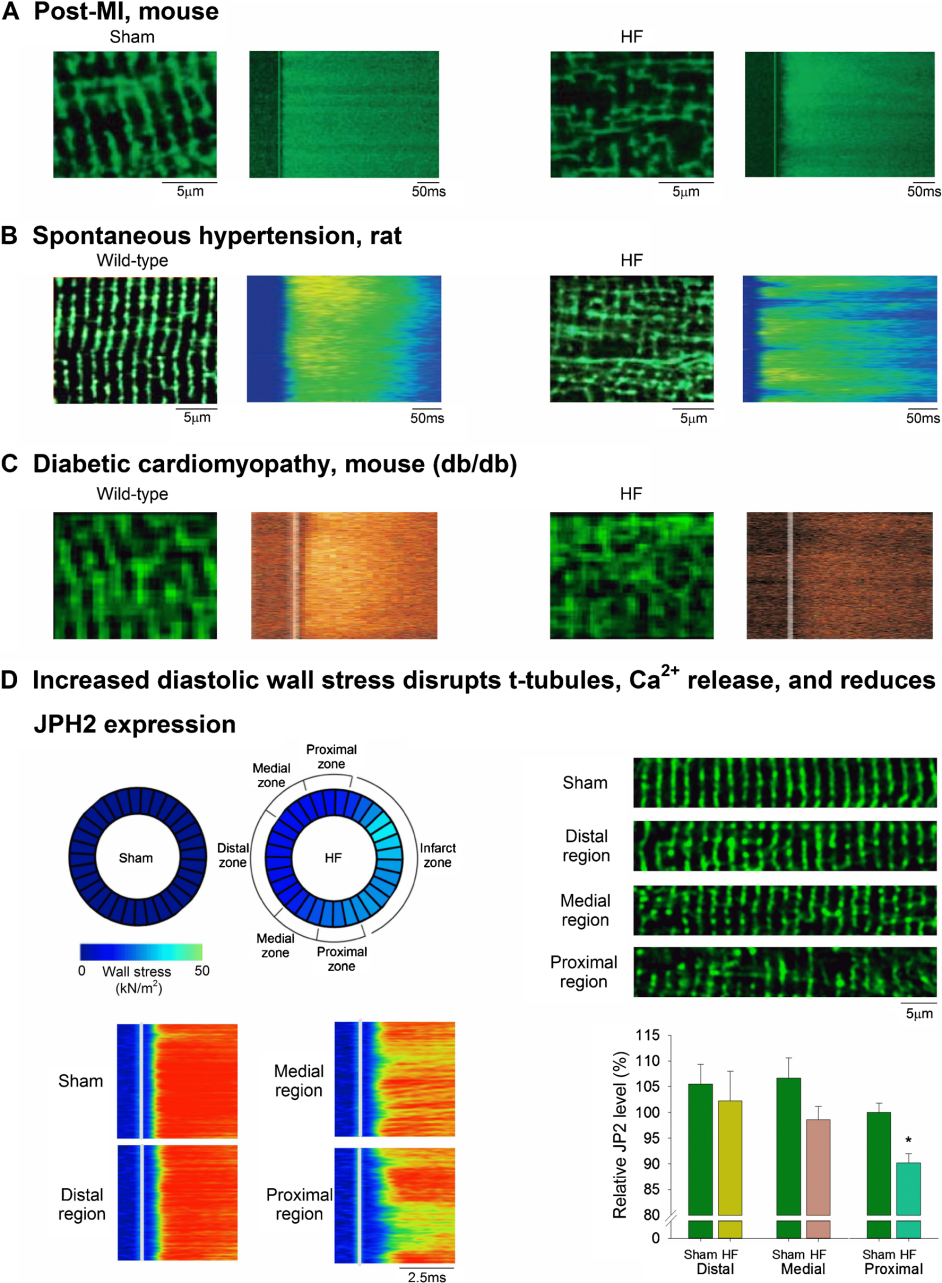
Structural and functional alterations in t-tubules during heart failure. **a–c** Confocal images of t-tubular structure (di-8-ANEPPS staining) in healthy and diseased ventricular cardiomyocytes. The well-organized t-tubule network observed in myocytes from wild-type and sham-operated hearts is lost and disorganized during heart failure resulting from myocardial infarction, hypertension, and diabetes. This structural remodeling results in de-synchronized Ca^2+^ release across the cell, as indicated by confocal line-scan images. Images are modified from **a**: [38]; **b**: [39]; **c**: [62]. **d** Elevated ventricular wall stress leads to t-tubule disruption. In the post-infarction failing rat heart, high wall stress proximal to the infarct is associated with regional reduction of JPH2 expression, t-tubule loss and dyssynchrony of Ca^2^ release (modified from [63•]). All images reproduced with permission. [39] Copyright (2006) National Academy of Sciences

Further evidence of t-tubule malleability has come from studies of cardiac pathology. A large body of evidence from a number of research groups [38–40, 49–57] has indicated that heart failure is associated with marked t-tubule reorganization in both human patients and animal models (Reviewed in [58–61]). Although the specific etiologies underlying heart failure have varied across these studies, spanning chronic ischemia, infarction, aortic stenosis, diabetes, and dilated cardiomyopathy, remarkably similar changes in t-tubular structure are reported [58, 59]. These alterations include reduction in t-tubule density, an increased fraction of longitudinally-oriented tubules, and t-tubule dilation (Fig. 3 **A**–**C**). Interestingly, t-tubule reorganization appears to not be restricted to left ventricular myocytes during heart failure, as similar findings have been reported in the right ventricle [3, 64] and in atrial cells [5, 15, 16, 65, 66]. Comparable atrial t-tubule reorganization resembling that reported in heart failure has been reported in atrial fibrillation [15, 16]. Thus, an emerging narrative indicates that there is impressive plasticity of t-tubule structure in all chambers of the heart, which is manifested across a range of cardiac pathologies.

As in developing cells, a low density of poorly organized t-tubules promotes de-synchronized and protracted Ca^2+^ release in failing cardiomyocytes (Fig. 3 **a**–**c**) [38, 39, 43]. This slowed Ca^2+^ transient has been coupled to the slowed and decreased amplitude of contraction that is a hallmark of the failing heart [67, 68]. Indeed, time course studies have indicated that t-tubule disruption occurs prior to the development of heart failure, suggesting that changing t-tubule structure may play a causative role in driving disease progression [3, 57]. Importantly, beyond the overt alterations in t-tubule structure, smaller dyadic-scale changes are also suggested to contribute to de-synchronization of Ca^2+^ release, including dispersion of RyR clusters which slows Ca^2+^ sparks [69]. Loss of various t-tubule-localized membrane currents can also de-synchronize Ca^2+^ release by re-shaping the action potential [70–72]. Furthermore, the impaired force-frequency response, characteristic of failing cells, may be related to t-tubule loss. As pacing frequency increases, a marked elevation of intracellular Na^+^ levels occurs which, via NCX, leads to gain of SR Ca^2+^ content and release. Removal of NCX along with t-tubules diminishes this response [73].

In addition to negative effects of t-tubule reorganization on contractility in the failing heart, altered t-tubule structure is believed to have rather complex consequences for arrhythmia generation (reviewed in [74]). Delayed afterdepolarizations (DADs) result from spontaneously released Ca^2+^ being extruded by NCX. For reasons which are not entirely clear, Ca^2+^ sparks occur almost exclusively at intact dyads where t-tubules are present [27], and several studies have reported low Ca^2+^ spark generation at orphaned RyRs [69, 75]. Furthermore, if an orphaned RyR cluster does generate a Ca^2+^ spark, DAD generation will be minimized since NCX is not located nearby [33, 76]. On the other hand, upon generation of a full-blown Ca^2+^ wave, fewer nearby NCX proteins in t-tubules will mean that less Ca^2+^ is pulled away from the developing wave front, making its propagation more likely. T-tubule reorganization during heart failure may have similarly complex and opposing effects on the generation of early afterdepolarizations (EADs). Loss of Ca^2+^ and NCX currents along with t-tubules is expected to shorten the action potential, making phase 2 EADs less likely but phase 3 EADs more likely [74, 76]. Shorter action potentials and shorter refractory period additionally increase the likelihood of re-entry. Thus, although this remains a developing field, there is certainly evidence to suggest that therapeutic t-tubule protection and repair, discussed in the following section, may benefit inotropy while inhibiting arrhythmia in the failing heart.

## T-Tubule Regulators—Opportunities for Therapy

The striking similarity between t-tubule structure in developing and failing cardiomyocytes has led to speculation that t-tubule disruption during heart failure may be linked to re-emergence of the fetal gene program in this disease [48]. Indeed, recent data identifying molecular regulators of t-tubule structure indicate that the late stages of dyadic assembly are likely amongst the first to be reversed during heart failure development. Although our understanding of the common signals underlying these disparate conditions remains in its infancy, once recognized, manipulation of these pathways could be harnessed for novel disease treatments. Such approaches may aim to safeguard t-tubular structure, or possibly grow new dyads [77]. Understanding the signaling pathways controlling t-tubule structure and growth is additionally hoped to have applications for the therapeutic maturation of cardiac stem cells, as at present these cells retain a quite immature phenotype [78, 79]. However, even before a full comprehension of t-tubule-controlling signals is attained, therapeutic intervention may be possible through manipulation of players already shown to be involved in dyadic assembly. While not necessarily addressing the root causes of dyadic breakdown, modulation of these players has shown early promise at the pre-clinical level, as outlined below.

### Junctophilin-2

Junctophilin-2 (JPH2) is a structural membrane protein that anchors the sarcolemma to the SR [80–83] (Fig. 2). This protein is characterized by a cytoplasmic MORN (membrane on receptor nexus) motif which affixes to the sarcolemma and a transmembrane domain embedded in the SR. The critical role of JPH2 in forming dyads is supported by the parallel appearance of JPH2 and t-tubules along z-lines during development [44]. Furthermore, JPH2 knockdown prevents full t-tubule maturation, leading to heart failure in developing mice [84, 85]. It appears that JPH2 is similarly important for maintaining established dyads in adult cardiomyocytes, as JPH2 downregulation during heart failure has been linked to t-tubule remodeling [3, 54, 63•, 64, 86, 87]. Recent data from our laboratory showed further evidence of pairing of JPH2 expression and t-tubule organization across failing, post-infarction hearts, with the most marked loss of JPH2 and t-tubules observed in regions neighboring the infarct [63•]. Of note, JPH2 has been suggested to anchor transverse but not longitudinal elements of t-tubules [84], consistent with the observation that decreased JPH2 expression during heart failure is associated with an increasingly longitudinal t-tubule orientation. Importantly, while some longitudinal elements present in failing cells may be those which have become unthethered and drifted following JPH2 loss, others are newly grown, and this can occur even in the presence of an intact transverse tubule population [22].

More detailed insight into the role of JPH2 in dyadic organization has been provided by nanoscale super-resolution imaging techniques. Munro and colleagues recently reported that JPH2 overexpression resulted in the formation of larger RyR clusters within CRUs [88]. In apparent support of a role for JPH2 in localizing dyadic RyRs, Wang *et al.* observed reduced co-localization of RyRs with NCX following JPH2 knockdown [31]. Exciting recent data suggest that JPH2 binding might not only localize RyRs to the dyad, but also stabilizes channel function, as RyR hyperactivity has been reported following JPH2 knockdown [89]. Similarly, a mutation of JPH2 (E169K) which reduces its binding to RyR was observed to increase RyR opening (leak) [90], while JPH2 overexpression was associated with inhibition of Ca^2+^ sparks [88]. Other proposed regulatory roles of JPH2 in the dyad include modulation of LTCCs, as reported previously in skeletal muscle [91] and recently in heart [92].

The precise mechanisms responsible for promoting JPH2 downregulation during heart failure continue to be examined. Data from our group indicate that elevated ventricular wall stress is an important trigger of JPH2 suppression (discussed below, [63•]), and while we did not identify the intermediate signaling pathway, interesting data from the Wang group have implicated a key role of miR-24 upregulation [93, 94]. Recently reported calpain cleavage of JPH2 [95] may also be expected to be augmented in the failing heart.

As there is now considerable evidence linking JPH2 downregulation to impaired Ca^2+^ homeostasis and arrhythmia in heart failure, this protein is considered to be a promising therapeutic target. JPH2 overexpression has been observed to restore t-tubule structure and abnormal SR Ca^2+^ release in failing cardiomyocytes, attenuating disease progression [96, 97•]. A putative alternative strategy for protecting JPH2 expression in the failing heart is via inhibition of miR-24, and Li and colleagues observed that miR-24 suppression did indeed prevent transition from compensated to decompensated hypertrophy [94]. However, miR-24 is also reported to play a key role in protecting the heart against apoptosis following ischemia [95], suggesting that miR-24 inhibition may not be a suitable treatment for all heart failure etiologies. As yet untested strategies for protecting JPH2 levels in the failing heart include blockade of JPH2 cleavage by calpain [98], or other degradation pathways. While these ideas are promising, it is important to note that present data supporting a potential therapeutic role of JPH2 in heart failure come almost exclusively from small rodents. Thus, future investigations will certainly require advancement to larger animal models to demonstrate possible relevance for human patients.

### Bridging Integrator-1 (BIN1)

The membrane scaffolding protein BIN1, or amphiphysin-2, plays an important role in dyadic assembly and maintenance [99–101]. The protein is expressed in several tissue specific isoforms. While the skeletal muscle isoform has long been known to induce tubulogenesis in this tissue [100, 102], the precise roles of the cardiac isoform have been recognized more recently. Four different splice variants are expressed in the mouse heart, and have been named according to their included exons: BIN1 (excluding exons 7, 11, and 13–17), BIN1+17 (including exon 17), BIN1+13, and BIN1+13+17 [103•]. BIN1+13 is the most abundant of these variants and is primarily involved with cell proliferation [104]. BIN1+13+17 on the other hand, promotes t-tubular growth, and folding of the membrane to create microdomains [101, 103•, 105]. These microdomains are believed to be the target for the previously demonstrated trafficking of LTCCs along microtubules to the t-tubule membrane [101]. In addition to facilitating membrane folding, BIN1+13+17 also anchors the growing end of LTCC-transporting microtubules in a process known as targeted delivery [103•]. The same isoform has been shown to attract phosphorylated RyRs on the SR membrane, presumably to ensure close proximity with dyadic LTCCs [106].

Consistent with a key role of BIN1 in forming and sustaining dyads, genetic knockout was observed to be embryonically lethal, and cardiomyocyte-specific deletion to promote dilated cardiomyopathy [107]. Downregulation of BIN1 has been reported in both human and animal models of heart failure, and associated with loss of t-tubules [54, 92, 108, 109]. The mechanism by which BIN1 loss leads to t-tubule membrane degradation is not clear, but may include its role in regulation of phosphoinositides. BIN1 clusters phosphoinositides during t-tubule formation, leading to dynamin-2 polymerization [100], and disruption of the phosphoinositide system has been linked to t-tubule degradation in both the heart [110] and skeletal muscle [111]. Key players appear to include myotubularin, a phosphoinositide-3 phosphatase [112], and phosphoinositide 3-kinases [110].

The consequences of BIN1 loss during heart failure are proposed to include macroscale loss of t-tubule membrane and functional dyads, with expected negative consequences for EC coupling efficiency. However, nanometer scale alterations may also be critical. Hong *et al.*, have suggested that t-tubule dismantling includes a less folded t-tubule membrane which increases arrhythmia susceptibility by allowing augmented ionic diffusion within the t-tubule lumen [103•]. Furthermore, BIN1’s reported role in trafficking LTCCs and RyRs to the dyad may contribute to unpacking of these proteins when BIN1 levels decline during heart failure progression.

Given the role of BIN1 in organizing t-tubule membrane folds and dyads it may be a potential target for future therapy. While existing data suggest that increasing BIN1 levels in heart failure might simultaneously augment contractility and attenuate arrhythmogenesis, such studies have not yet been conducted. However, given what appears to be rather complex interaction between BIN1 and a variety of lipid and protein partners, therapeutic manipulation of this system may not be straight forward.

### Load

Accumulating evidence supports a key role of workload as a dynamic regulator of t-tubular structure (reviewed in [113]). While increased workload during heart failure has been linked to t-tubule disruption in a large number of studies, it appears that such remodeling is reversible upon unloading of failing hearts. The Terracciano group has demonstrated this point by performing heterotopic transplantation of hearts from failing rats [113]. Similar findings have been made with pharmacological interventions that unload the failing heart, such as sildenafil and β_1_-receptor blockers [64, 86, 114]. Similarly, resynchronization therapy has been observed to improve cardiac function by restoring the t-tubule network in dyssynchronous heart failure [115]. Interestingly, Ibrahim and colleagues observed that unloading *healthy* hearts promoted t-tubule loss [116]. Thus, these authors have proposed that there is an optimal range of loads which is necessary to maintain t-tubules [113].

Workload is of course a rather nonspecific term, which may encompass many aspects of cardiac function. In an effort to more specifically identify signals which relate cardiac workload to t-tubular remodeling, we recently examined the role of ventricular wall stress [63•]. We found that wall stress was negatively correlated with t-tubule density across the post-infarction failing heart, with a marked elevation of wall stress and loss of t-tubules observed in regions neighboring the infarct (Fig. 3D). In situ experiments confirmed a causal nature of this relationship, as stretching papillary muscles to reproduce high, in vivo levels of wall stress signaled t-tubule degradation [63•]. While the underlying mechanism includes JPH2 downregulation, the critical stretch/wall stretch sensors which detect and relay these signals remain to be determined. One promising candidate is the z-disc protein titin cap (Tcap). Tcap loss has been demonstrated during heart failure [55, 108] and Tcap knockout mice have been observed to exhibit progressive disruption of the t-tubule network during development [117]. Augmented expression of Tcap, on the other hand, is associated with recovery of t-tubules, for example during reverse remodeling induced by SERCA2a gene therapy [108].

Workload-dependent regulation of t-tubules has important therapeutic implications. As noted above, this mechanism has already been linked to the benefits of existing load-reducing drugs, but also likely contributes to the benefits of therapies such as ventricular assist devices [113]. Quantitative understanding of how workload parameters, such as ventricular wall stress, regulate t-tubule morphology could be envisioned to guide the application of such therapies. Future treatment strategies might alternatively directly inhibit the mechanosensing that signals t-tubule remodeling, although at present our understanding of these pathways is limited to only a couple putative players (Tcap, JPH2). Intriguing recent data suggest that the t-tubules themselves can transduce mechanical signals during stretch and contraction [118, 119] and possibly thereby regulate their own structure. If true, t-tubule membrane malleability might be clinically targeted, in an effort to perhaps counteract stiffening of the t-tubule lumen due to collagen deposition (Crossman *et al.*, Cardiovasc Res [120]).

## Conclusion

The above discussion has highlighted the impressive plasticity of cardiomyocyte t-tubular structure, and its central importance in regulating cellular Ca^2+^ homeostasis, contractility, and arrhythmic potential. While loss and disorganization of t-tubules is now understood to be an important cellular-level contributor to heart failure, the malleability of this system may also be harnessed for therapy. Potential therapeutic targets include JPH2 and BIN1, which are critical for dyad formation and maintenance, and are downregulated in the failing heart. As recent data indicate that expression of these dyadic stabilizers is at least in part controlled by workload, future therapies may act to more effectively unload the failing heart and/or disrupt mechanotransduction.

## Abbreviations

BIN1: bridging integrator-1
CICR: Ca^2+^-induced Ca^2+^ release
DAD: delayed afterdepolarization
EAD: early afterdepolarization
EC coupling: excitation-contraction coupling
JPH2: junctophilin-2
LTCC: L-type Ca^2+^ channel
RyR: ryanodine receptor 2
SR: sarcoplasmic reticulum
NCX: sodium-calcium exchanger 1
Tcap: titin cap
